# Modeling Curvature-Dependent Subcellular Localization of the Small Sporulation Protein SpoVM in *Bacillus subtilis*

**DOI:** 10.1371/journal.pone.0111971

**Published:** 2015-01-27

**Authors:** Vaibhav Wasnik, Ned S. Wingreen, Ranjan Mukhopadhyay

**Affiliations:** 1 Department of Physics, Clark University, Worcester, Massachusetts, United States of America; 2 Department of Molecular Biology, Princeton University, Princeton, New Jersey, United States of America; University of Illinois at Urbana-Champaign, UNITED STATES

## Abstract

Recent *in vivo* experiments suggest that in the bacterium, *Bacillus subtilis*, the cue for the localization of the small sporulation protein, SpoVM, an essential factor in spore coat formation, is curvature of the bacterial plasma membrane. *In vitro* measurements of SpoVM adsorption to vesicles of varying sizes also find high sensitivity of adsorption to vesicle radius. This curvature-dependent adsorption is puzzling given the orders of magnitude difference in length scale between an individual protein and the radius of curvature of the cell or vesicle, suggesting protein clustering on the membrane. Here we develop a minimal model to study the relationship between curvature-dependent membrane adsorption and clustering of SpoVM. Based on our analysis, we hypothesize that the radius dependence of SpoVM adsorption observed *in vitro* is governed primarily by membrane tension, while for *in-vivo* localization of SpoVM, we propose a highly sensitive mechanism for curvature sensing based on the formation of macroscopic protein clusters on the membrane.

## Introduction

Recent advances in fluorescence microscopy [1] have revealed a surprising degree of protein organization and segregation on bacterial membranes [2]. Proteins are found to localize to regions such as mid-cell planes and poles of rod-shaped bacteria that were not thought to be chemically distinct. Understanding the mechanisms for localization, in the absence of vesicle-mediated sorting machinery such as found in eukaryotic cells, has become a fundamental challenge in bacterial cell biology [3]. An intriguing example of sub-cellular localization occurs during spore formation when the protein SpoVM (VM) localizes to a region of the plasma membrane in the bacterium *Bacillus subtilis*. The localization of VM to the outer membrane surrounding the forespore is believed to be the first step in the formation of a complex protein shell consisting of more than 70 proteins that are produced in the mother cell and deposited around the spherical spore to create the spore coat [4, 5]. How does VM discriminate between the spore membrane and the cytoplasmic membrane of the mother cell? Ramamurthi *et al.* [6] recently suggested that VM recognizes a geometric cue, namely membrane curvature, thereby discriminating between the concave shape of the cytoplasmic membrane and the convex shape of the outer forespore membrane. To explore the dependence of VM binding on membrane curvature, the authors prepared spherical vesicles varying in radius from 1 to 30 *μ*m, incubated these with purified VM solution, and found a pronounced dependence of VM adsorption on membrane curvature (as determined by vesicle radius). Furthermore, dependence of adsorption on SpoVM concentration suggested that protein clustering might play an important role in curvature-dependent localization.

**Figure 1:**
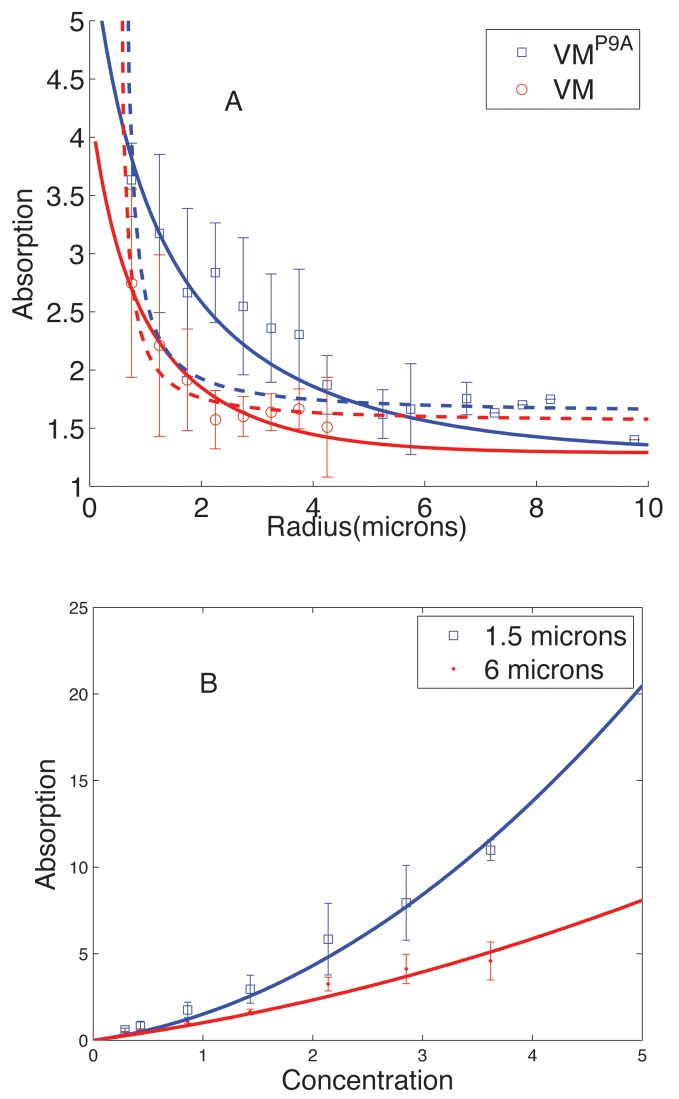
Experimental results from Ramamurthi *e*t al. [6] on SpoVM adsorption on vesicles of varying radii, *r*, (Experimental data, courtesy: Kumaran Ramamurthi). A) SpoVM adsorption as a function of vesicle radius. Data points correspond to experimental results for wild-type VM (open circles) as well as VM^P9A^ (open squares). The data has been fit to two models (see text): (i) Curvature model - the dashed curves are the best fits with a repulsive interaction of the form *e*_*r*_ = *c*_1_ − *c*_2_/*r* where *c*_1_ = 2.67*k*_*B*_ *T*, *c*_2_ = 1.1649*k*_*B*_ *Tμ*m for VM and *c*_1_ = 2.42*k*_*B*_ *T* and *c*_2_ = 1.2277*k*_*B*_ *Tμ*m for VM^P9A^. Here *k*_*B*_ stand for the Boltzmann constant and *T* is room temperature (300 K). (ii) Surface tension model - the solid curves are best fits with a repulsive interaction of the form *e*_*r*_ = *c*_1_ + *c*_2_ * *r* where *c*_1_ = .7*k*_*B*_ *T* and *c*_2_ = .61*k*_*B*_ *Tμ*m^−1^ for VM, and *c*_1_ = .5366*k*_*B*_ *T* and *c*_2_ = .34*k*_*B*_ *Tμ*m^−1^ for VM^P9A^. B) Data and best fit curves to SpoVM adsorption (in arbitrary units) versus concentration in solution data for vesicle radii 0.75 *μ*m and 3 *μ*m.

VM is a small protein of 26 amino acids, structurally an amphipathic alpha helix around, or less than, 4 nm in length [6]. VM inserts into a phospholipid bilayer possibly in such a way that its long axis is parallel to the membrane, with the hydrophobic face buried in the lipid tails and the hydrophilic (in this case, charged) face exposed to water. Interestingly, substituting a single proline at position 9 to alanine (VM^P9A^) results in indiscriminate mislocalization of the protein. The strong curvature dependence of VM membrane adsorption, as depicted in Fig. 1A for the *in vitro* experiments, is puzzling considering the orders of magnitude difference between the length scale of individual proteins (4 nm) and the radii of membrane curvature (0.5 *μ*m). As noted in Refs. [3, 6], for a 4 nm rod lying flat on the surface of a 1 *μ*m diameter sphere the maximum distance between one end of the rod and the surface of the sphere is less than 0.2 $\dot{A}$. However, adapting the arguments from [7, 8], a cluster of proteins could more readily differentiate such small curvatures due to the cluster’s larger overall size. Thus, the strong dependence of VM adsorption on the radius of the vesicle might indicate clustering of VM on the membrane.

Support for the role of clustering comes from the dependence of adsorption on VM concentration in solution [6] as shown in Fig. 1B. At low concentrations, VM adsorption is linear and approximately independent of vesicle curvature, suggesting that at low concentration VM adsorption is non-cooperative and that binding of individual proteins to the membrane is independent of membrane curvature under the given conditions. At higher concentrations, for radii *r* larger than a critical radius, *r*_*c*_ ≈ 2*μ*m, VM adsorption continues to increase linearly with concentration. In contrast, for *r* < *r_c_* the adsorption curves become steeper at larger concentrations, suggesting cooperative binding of VM to the membrane, consistent with clustering of membrane-associated VM.

In this paper, we develop and study a minimal statistical mechanical model of membrane-bound protein clustering to investigate further the relationship between clustering and localization, and to analyze the results presented in [6]. In our model, we include both short-range attractive interactions between membrane-associated VM that drive clustering, as well as longer-range repulsive interactions that limit cluster size. The short-range attractive interactions could arise from direct chemical interactions between amino-acid side chains or could be membrane mediated, arising for example from the hydrophobic mismatch between the hydrophobic side of the helix and the thickness of the lipid bilayer or leaflet [9]. Longer-range repulsive interactions could arise from elastic deformations that the proteins impose on the membrane. For example, if VM proteins in the membrane asymmetrically deform the head and tail regions of the outer leaflet, as has been suggested in [3], they would effectively act as conical or wedge-shaped inclusions; the emergence of membrane mediated repulsive interactions between such inclusions has been demonstrated [10, 11]. Inclusion of repulsive interactions is essential for our model. In its absence we would expect a sharp transition from a sparsely covered membrane to a state where the VM molecules would essentially form a giant cluster implying a step like jump in the adsorption as a function of concentration that is not seen in the experimental data (Fig. 1B). The interplay between short-range attraction and longer-range repulsion could stabilize clusters of some characteristic finite size, as has been demonstrated theoretically and experimentally [8, 12–21]. In the context of SpoVM adsorption, formation of membrane associated finite-sized clusters can account for the deviation from linearity in adsorption versus concentration exhibited in Fig. 1B.

## Clustering Model

**Figure 2:**
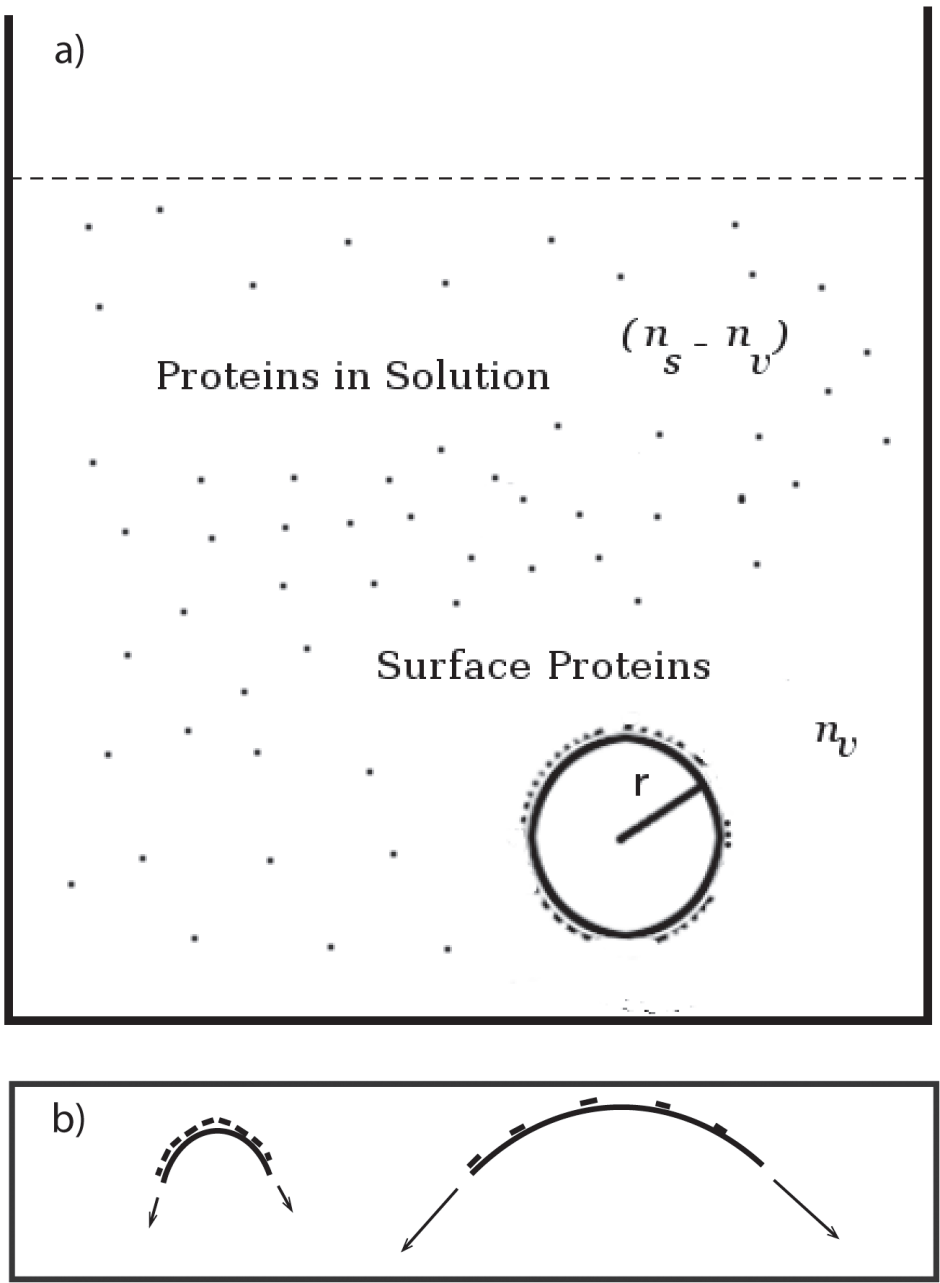
Schematic of spherical vesicle in protein solution. a) Schematic depiction of vesicle of radius *r* in bath of SpoVM molecules; *n*_*s*_ is the total number of VM molecules in solution and *n*_*v*_ is the total number of VM molecules on the surface of the vesicle. b) Two vesicles of different radii with a higher concentration of VM molecules on the surface of the larger vesicle. We suggest in the paper that the larger vesicle also has a higher membrane tension and that SpoVM is detecting membrane tension rather than curvature directly in the *in-vitro* experiments.

In order to analyze the experiments of VM adsorption to vesicles of varying diameters [6], we developed a minimal thermodynamic model of VM clustering and adsorption. We consider a vesicle of radius *r* in contact with a bath of VM molecules in a dilute solution; in Fig. 2a), we show a schematic of the system. For convenience and simplicity, we represent the vesicle membrane and the surrounding solution as a lattice where each lattice site could be occupied by a SpoVM molecule (or be empty), though our results are general and not tied to such details of the model. We represent the solution as a lattice with *L*_*s*_ sites, and let *n*_*s*_ be the total number of VM molecules (in solution as well as on the vesicle). Let *n*_*v*_ be the number of VM molecules on the surface of the vesicle, and *L*_*v*_ (≈ 4*r* ^2^/*a* ^2^) be the number of lattice sites on the surface of the vesicle, where *a* is a length corresponding to an effective SpoVM radius. In the limit where *n*_*s*_ is much greater than *n*_*v*_, the ratio *n*_*s*_/*L*_*s*_ is proportional to the concentration *c*_*s*_ of VM in solution.

We assume that in solution the VM molecules exist as monomers but can cluster on the vesicle membrane due to inter-protein short-range attractive forces. The total energy *E* has three contributions: (i) the binding energy, *ϵ*_*b*_, of a protein to the membrane, (ii) a short-range attractive interaction between membrane-bound VM units (modeled via an interaction energy *ϵ*_*nn*_ between a protein and all its nearest neighbors), and (iii) a longer range repulsive interaction between membrane bound VM units arising, for example, from the elastic packing stresses in the membrane. Initially, we assume that the range of repulsive interactions is larger than the cluster size so that within a cluster each protein interacts with every other protein with equal strength [8]. For a cluster of size *k*, the energy contribution due to repulsive interactions is given by *ϵ*_*r*_ *k*(*k* − 1)/2 where *ϵ*_*r*_ characterizes the strength of repulsive interactions. The total energy can thus be written as

$$E=-\epsilon_{b}n_{v}+\sum_{k}\left[-\left(k-1\right)\epsilon_{nn}+\frac{1}{2}k\left(k-1\right)\epsilon_{r}\right]m_{k},$$ (1)

where *m*_*k*_ denotes the number of clusters of size *k*.

Thermodynamic equilibrium of the systemm is characterized by the minimum of the free energy *F* = *E* − *TS*, where *T* is the temperature and *S* is the entropy. In the dilute limit, the entropy is given by [22]

$$\begin{array}{ccc} S & \approx & k_{B}\ln\frac{L_{s}!}{\left(L_{s}-n_{s}+n_{v}\right)!\left(n_{s}-n_{v}\right)!}\\ & & +k_{B}\ln\frac{L_{v}!}{\left[\Pi_{k}m_{k}!\right]\left(L_{v}-n_{v}\right)!}, \end{array}$$ (2)

noting that *n*_*v*_ = ∑_*k*_ *km*_*k*_. In order to minimize the free energy *F*, we set $\frac{\partial (E-TS)}{\partial m_{k}}$ to zero, and using Stirling’s approximation, we find

$$k_{B}T\left[k\ln\frac{L_{s}-n_{s}+n_{v}}{n_{s}-n_{v}}+\ln\frac{m_{k}}{L_{v}-n_{v}}\right]+\frac{\partial E}{\partial m_{k}}=0.$$ (3)

Assuming *n*_*v*_ ≪ *L*_*v*_, *n*_*s*_, we can write this as

$$k_{B}T\left[k\ln\frac{1-n_{s}^{*}}{n_{s}^{*}}+\ln\frac{m_{k}}{L_{v}}\right]+\frac{\partial E}{\partial m_{k}}=0,$$ (4)

where $n_{s}^{*}=n_{s}/L_{s}$ represents the concentration of proteins in the bath. Hence,

$$n_{v}=\sum_{k}km_{k}=\sum_{k}kL_{v}{\left(\frac{n_{s}^{*}}{1-n_{s}^{*}}\right)}^{k}e^{-\frac{\partial E}{\partial m_{k}}/k_{B}T}$$ (5)

In the dilute limit, *n* ^*^_s_ ≪ 1, we can approximate

$$n_{v}=\sum_{k}kL_{v}{\left(n_{s}^{*}\right)}^{k}e^{-\frac{\partial E}{\partial m_{k}}/k_{B}T}.$$ (6)

Substituting Eq. 1 for the energy, and noting that the protein concentration $c_{s}=\alpha n_{s}^{*}$, where *α* is a constant, we obtain

$$n_{v}=L_{v}e^{-\epsilon_{nn}/k_{B}T}\sum_{k}k\;e^{-(\frac{k\left(k-1\right)\epsilon_{r}}{2k_{B}T})}\;{\left(\frac{c_{s}}{c_{0}}\right)}^{k}$$ (7)

where *c*_0_ = *e* ^−(*ϵ*_*nn*_+*ϵ*_*b*_)/*k*_*B*_*T*^ *α* is a parameter of the model.

If we define the normalized concentration of proteins in the solution, *C_s_* = *c_s_*/*c*_0_ and the normalized concentration of proteins bound to the vesicle $C_{v}=n_{v}/(L_{v}e^{-\epsilon_{nn}/k_{B}T})$ and *e_r_* = ϵ*_r_*/*k_B_T* we can write

$$C_{v}=\sum_{k}k\;e^{\frac{-k\left(k-1\right)e_{r}}{2}}\;{\left(C_{s}\right)}^{k}.$$ (8)

Note that this equation is generally valid in the dilute limit and not tied to the lattice model. We evaluate the sums numerically using MATLAB.

In the limit of low concentration, that is *C*_*s*_ ≪ 1, the sum is dominated by the first term *k* = 1 which is independent of the repulsive energy *e*_*r*_. This indicates that for small *C*_*s*_ most vesicle-bound proteins exist as monomers and that the concentration *C*_*v*_ of membrane-bound proteins is proportional to the concentration *C*_*s*_ of proteins in solution. On the other hand, for large *C*_*s*_ we expect the leading contribution to come from the term with the largest magnitude in Eq. 8, corresponding to *k* = *k* ^*^ where *k* ^*^ ≈ (ln *C*_*s*_)/*e*_*r*_. In this limit, *C*_*v*_ is approximately given by

$$\ln C_{v}\approx \frac{{\left(\ln C_{s}\right)}^{2}}{2e_{r}},$$ (9)

assuming the solution is still in the dilute limit and protein adsorption on the vesicle surface has not reached saturation.

## Results and comparison to experiments

**Figure 3:**
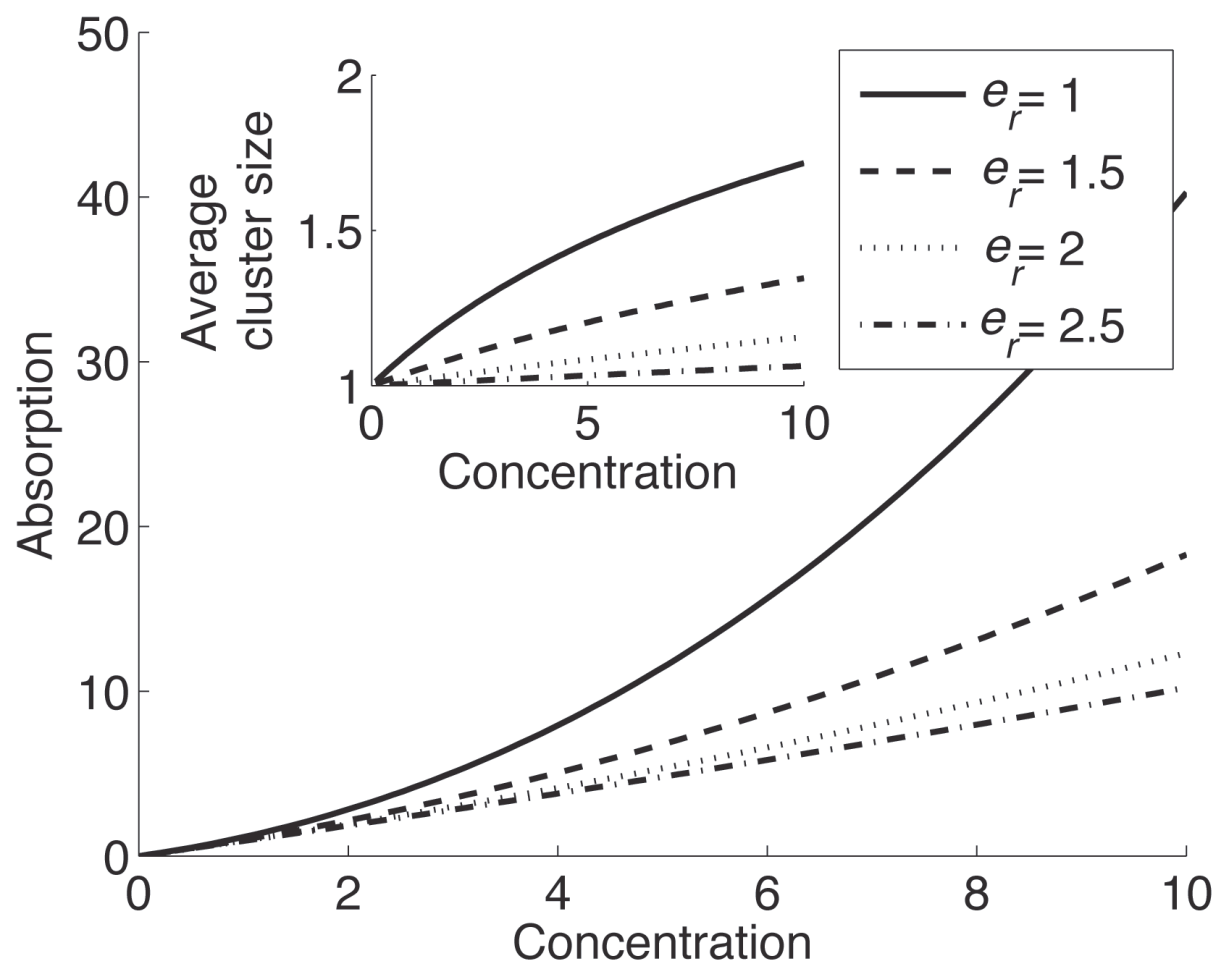
Dependence of SpoVM adsorption on the strength of repulsive interaction *e*_*r*_. Adsorption (as characterized by the concentration of vesicle-bound proteins *C*_*v*_) versus SpoVM concentration in solution for different values of *e*_*r*_ from Eq. 3. (Inset) Average cluster size versus SpoVM concentration for different values of *e*_*r*_.

Our aim in this paper is to model the curvature dependence of SpoVM membrane adsorption. Experiments find no significant dependence of SpoVM adsorption on vesicle radius at low concentrations [6]. Theoretically, in the limit of low protein concentration where the expression for *n*_*v*_ in Eq. 6 is dominated by the *k* = 1 term, $n_{v}\approx n_{s}^{*}L_{v}e^{\epsilon_{b}/k_{B}T}$, where $n_{s}^{*}$ represents VM concentration in solution, leading us to conclude that the binding energy *ϵ*_*b*_ is essentially independent of vesicle radius for the range considered (≥ 0.75 *μ*m). Since, as argued earlier, the origin of the repulsive interaction is likely to lie in stresses induced by the embedded proteins in the outer lipid leaflet, the repulsive energy *e*_*r*_ is the parameter expected to be most sensitive to changes in the membrane curvature. Correspondingly we will also take the short range interaction *ϵ*_*nn*_, and hence the constant *c*_0_, to be curvature independent. In Fig. 3, we plot VM adsorption versus VM concentration in solution at different values of the repulsive energy *e*_*r*_. As expected, at larger values of *e*_*r*_ the terms corresponding to large cluster size in Eq. 8 are suppressed and we find a linear relationship between *C*_*v*_ (∼ SpoVM adsorption) and solute concentration for the range shown. Also as expected at lower values of *e*_*r*_, we find large deviations from linearity. Comparing Fig. 3 to the experimental results in Fig. 1 suggests that VM has a lower repulsive interaction energy at higher curvatures. If, as argued earlier, the membrane-inserted helix effectively acts as a wedge-shaped inclusion, the origin of the repulsive interaction could lie in the mismatch between over-all membrane curvature and the inclusion-induced preferred curvature. Since the curvature mismatch is smaller at larger membrane curvatures, qualitatively we expected lower repulsion at smaller radius, exactly as seen in the experiments.

Since in the experiments the vesicle radius *r* is orders of magnitude larger than the protein dimension *a*, we expect *e*_*r*_ can be expanded in powers of *a*/*r* (we expect to hold for any detailed physical model of curvature sensing). To linear order this expansion yields *e*_*r*_ = *c*_1_ − *c*_2_/*r*, where *c*_1_ and *c*_2_ are constants. Using this form for *e*_*r*_, we fit the experimental data for both wild-type SpoVM and the mutant, VM^P9A^, as shown in Fig. 1A (dashed lines). Even though the fit for wild-type SpoVM might appear adequate, the parameters had to be chosen so that SpoVM adsorption diverges at slightly lower vesicle radius (around 0.5 *μ*m), which would be highly unphysical. Thus the fit to the data is problematic. For VM^*P*9*A*^ mutant, the best fit is completely inadequate even without considering this divergence. The reason for the poor fit can be understood in terms of the Boltzman factor exp[−*E*/(*k*_*B*_ *T*)] which determines the probability of SpoVM membrane binding. Since the radius dependence of the repulsive interaction goes as *c*_2_/*r*, the adsorption (which is proportional to binding probability) has an exp(*ρ*/*r*) dependence, where *ρ* is a constant, which does not match the radius dependence in the experimental data points. Adding higher order terms in the expansion does not rectify the situation. Thus an expansion of *e*_*r*_ in powers of curvature does not explain the data, which is puzzling.

**Figure 4:**
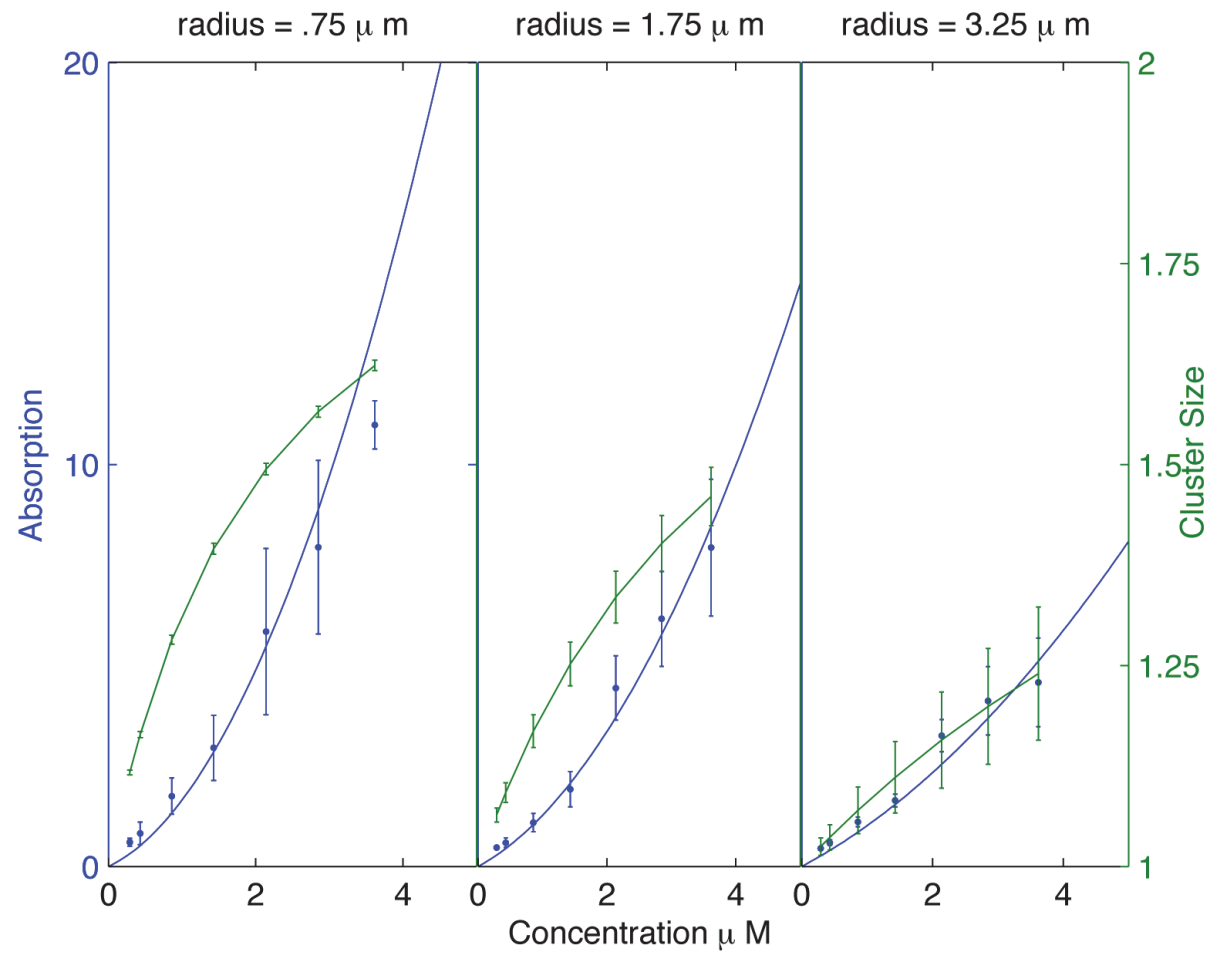
Membrane adsorption as a function of SpoVM concentration. We plot membrane adsorption (solid curve) and average cluster size (dashed curve) versus SpoVM concentration in solution using *e*_*r*_ = (0.7 + 0.6*r*)*k*_*B*_ *T*, for vesicle radii *r* = .75, *r* = 1.75, and *r* = 3.25 *μ*m. Based on experimental error bars, we have incorporated the appropriate error bars in the plots of cluster size, noting that cluster size is indicative of the degree of cooperativity in SpoVM adsorption. While still significant, the error bars are small enough to indicate relatively small cluster sizes with confidence.

We noticed that the data for radius dependence of SpoVM adsorption [6] (both for the wild-type and the mutant) is far more naturally fit by a form exp(−*br*), where *b* is a constant. Since adsorption is determined by the Boltzmann factor exp[−*E*/(*k*_*B*_ *T*)], this indicates the presence of a term in the energy which is proportional to the radius (rather than the curvature, i.e. 1/radius). Such a dependence can arise naturally from membrane tension, but seems incompatible with any direct curvature sensing mechanism. If, for example, the vesicles have approximately the same osmotic pressure difference across the membrane, given by *P*, then for the spherical vesicles the surface tension will be *σ* = *Pr*/2 ∝ *r*. It is known that repulsive membrane-mediated interactions between inclusions/embedded proteins can depend on the surface tension *σ*. Helfrich and coworkers [10] studied membrane-mediated interactions between conical inclusions and demonstrated that the resulting repulsive interaction strength depends on lateral surface tension. An expansion of the repulsive interaction energy in powers of surface tension, to linear order in surface tension *σ*, yields *e*_*r*_ ≈ *c*_1_ + *c̃*_2_ *σ* = *c*_1_ + *c*_2_ *r*, where *c*_1_, *c̃*_2_, and *c*_2_ are constant parameters independent of radius. Employing the form *e*_*r*_ = *c*_1_ + *c*_2_ *r*, we can satisfactorily fit the data for both VM and VM^P9A^ adsorption as a function of vesicle radius as well as the dependence of adsorption on VM concentration in solution (Figs. 1 and 4). The proposed dependence of SpoVM adsorption on surface tension also helps explain a puzzling feature of the experiments. For the best fit parameters from Fig. 4 we calculated the cluster size as a function of radius, and typically found average cluster sizes to be rather small, around 2–4 proteins, even for the smallest radius *r* = .75 *μ*m. From the viewpoint of any direct curvature sensing mechanism, it remains puzzling how such small clusters can detect differences in radius that are orders of magnitude larger. Thus, we propose that the *in vitro* data strongly suggests that in these experiments SpoVM is detecting membrane tension rather than the direct membrane curvature (see fig. 2b)). We note moreover that our model then suggests a higher degree of cooperativity involving larger clusters at lower values of membrane tension, which is likely to have consequences for *in vivo* localization, as discussed in the next section.

## Discussion and conclusions

Our analysis of the *in vitro* experiments [6] indicates that SpoVM molecules indeed cluster on the vesicle membrane and that cluster sizes are determined by the balance between short-range attractive and longer-range repulsive interactions. Moreover, our analysis suggests that the radius dependence of VM adsorption onto vesicles arises primarily from surface tension rather than curvature. We explicitly saw that a curvature induced repulsive interaction of the form *c*_1_ + *c*_2_/*r* could not fit the data for the SPoVM and its mutant VM^P9A^. However if the repulsive interaction is assumed to be surface tension mediated, leading to a repulsive interaction of the form *c*_1_ + *c*_2_ *r*, we can fit the experimental data very well. While in our model we assume non-stoichiometric clustering, where cluster sizes are determined by the balance between attractive and repulsive interactions, we cannot rule out the possibility that SpoVM forms small stoichiometric clusters of fixed size, say of size 3 monomers (see *Supplementary Material*, S1 File).

Our results predict that reduced surface tension will lead to higher SpoVM adsorption. The appreciable surface tension in the experiments balances the differences in osmotic pressures between the inside and the outside of the vesicle, because of the differences in molarity of the solutions. This balance would imply that the surface tension is given by *σ* ∼ *Pr*, where *P* is the osmotic pressure difference. Vesicle surface tension can be measured in a number of ways. One possibility is the measurement and analysis of membrane fluctuations [23], since membranes with lower surface tension would exhibit larger fluctuations. Using such methods it would be possible to quantitatively probe our predictions for adsorption versus surface tension.

Experiments where adsorption is measured at reduced vesicle surface tension, which can be achieved by changing the osmolarity of the surrounding solution, could also test our hypothesis. Presumably for *in vivo* SpoVM localization, SpoVM proteins do sense membrane curvature rather than surface tension since, prior to pinch-off, the forespore membrane is continuous with the cytoplasmic membrane of the mother cell and since we expect surface tension to be roughly uniform in the cytoplasmic membrane. Moreover, as reported in [6], SpoVM binds to the membrane indiscriminately in mutants arrested with a straight septum, and, in some fraction of mutants that develop a bulge, SpoVM localizes to the convex protrusion, indicating strongly that *in vivo* SpoVM senses membrane curvature. The bacterial plasma membrane is likely to have a relatively low surface tension due to excess plasma membrane (in comparison to the area of the enveloping cell wall) as indicated by membrane bulging when bacteria are exposed to antibiotics or lytic enzymes [22–24]. Since at lower surface tension we expect reduced strength of repulsion, our model predicts that SpoVM would form larger clusters that, due to the collective energy of the cluster, would be more effective at discriminating curvature.

**Figure 5:**
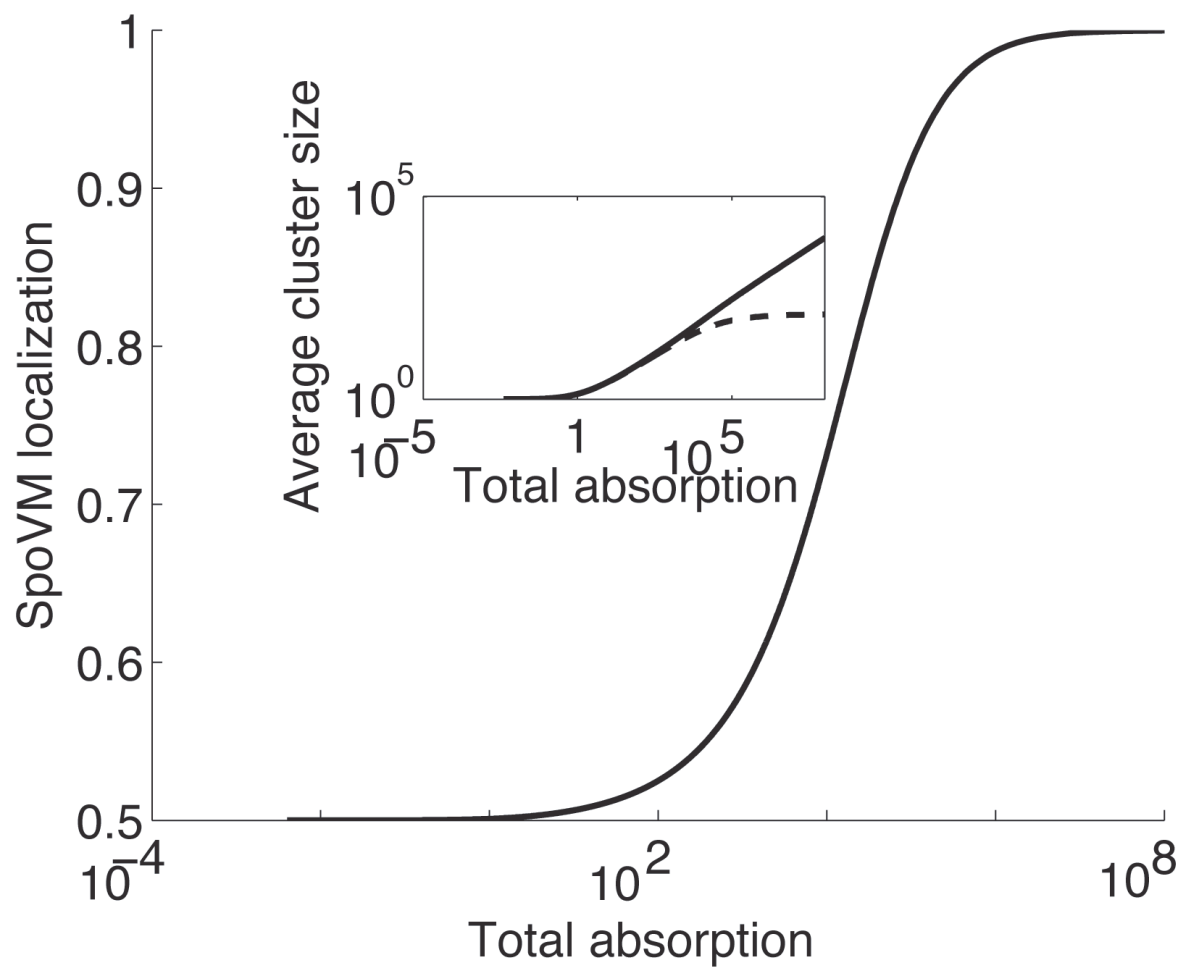
Differential SpoVM localization to region of higher curvature. SpoVM localization defined as SpoVM adsorption to membrane region with higher curvature divided by total adsorption is plotted against total SpoVM membrane adsorption based on a.01 relative difference in binding energy and identical repulsive interaction *e*_*r*_ = 0.2. The relatively small value of *e*_*r*_ is crucial for curvature-dependent localization. (Inset) Average cluster size on the two membrane regions are plotted against the total adsorption; the solid curve corresponds to cluster size on membrane region with higher curvature and the dashed curve corresponds to cluster size on the second membrane region.

**Figure 6:**
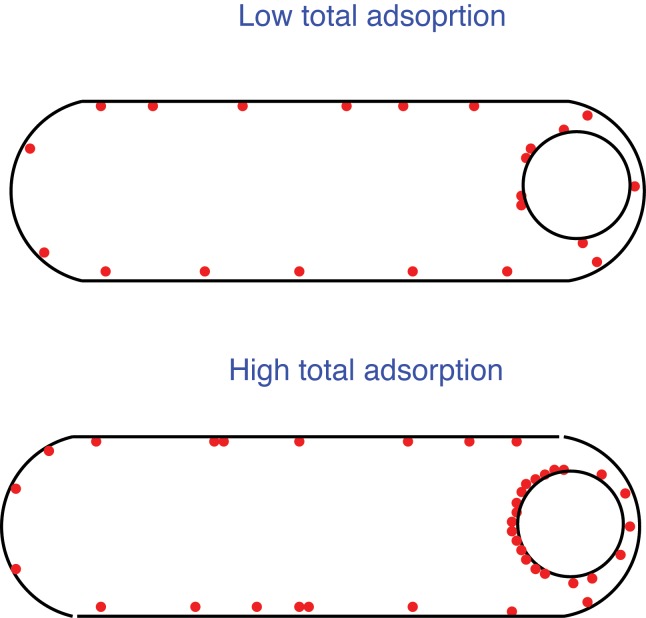
Schematic of the possible relation between clustering and curvature-based localization *in vivo* at low and high SpoVM concentration.

*In vivo*, prior to pinch-off, we do not expect any significant variation in the surface tension of the membrane, complicating the relation between the curvature dependence found *in vitro* and SpoVM localization to forespore *in-vivo*. How could a strong curvature dependent localization, as is exhibited *in vivo*, arise from a model like ours? We have assumed so far that all proteins in a cluster repel each other with equal strength, which should be reasonable for small clusters. However in reality we expect a finite range for the interaction, and clusters could ultimately grow bigger than this range. For simplicity let us assume that the repulsive interaction has the form of a step-function, the strength being a constant for inter-protein distance below a certain cutoff and zero when the distance becomes larger (a fair approximation to the gaussian profile derived in [8]). In this case the repulsive energy of a cluster would grow quadratically as *k*(*k* − 1)*ϵ*_*r*_/2 for cluster size *k* ≤ *k*_max_ and linearly as [*k*_max_(*k*_max_ − 1) + (*k* - *k*_max_)(*k*_max_ − 1)]*ϵ*_r_/2 for cluster size *k* > *k*_max_. In this case, low SpoVM concentration will result in small clusters, while above a critical SpoVM concentration most of the SpoVM will bind in one giant cluster. Now consider two membrane regions, region 1 and region 2 (for simplicity we assume they have the same area) with different curvatures. We expect that it will easier for a helix to insert into a convex shaped membrane, leading to a slightly greater binding energy *ϵ*_*b*_ for the region with higher curvature. For concreteness, assume that membrane region 1 has higher curvature and that *ϵ*_*b*_ in this region is just 1% larger than in the second region. In Fig. 5, we plot adsorption of SpoVM to membrane region 1 divided by total adsorption as a function of total adsorption (see S1 File for details of the underlying calculation). As expected, at low SpoVM concentrations, SpoVM does not discriminate between the two regions while at higher concentrations we observe a dramatic localization of SpoVM to the region of higher curvature(see Fig. 6 for a schematic).

Thus, more generally, we propose that SpoVM membrane adsorption depends both on membrane tension and on membrane curvature. Which effect dominates depends on the specific conditions. Our analysis suggests that for the *in vitro* measurements reported in [6], the strong dependence of adsorption on vesicle radius be explained in terms of tension sensing by SpoVM. On the other hand, *in vivo* the tension is expected to be approximately uniform throughout the membrane and thus localization can most readily be explained in terms of curvature sensing. As a corollary, this suggests that large clusters of SpoVM must form *in vivo* to enable sensing of curvature differences on micron length scales.

While in this paper we focus exclusively on SpoVM, exploitation of membrane curvature as a cue for subcellular protein localization appears to be widespread [3]. Two protein motifs have been extensively studied in this regards, amphipathic alpha helices [3, 27] and Bin-amphiphysin-RVS (BAR) domains [28]. While curvature sensing by amphipathic alpha helices occurs in both eukaryotic and prokaryotic cells, BAR domains appear exclusively in eukaryotes. In eukaryotic systems, the ArfGAP1 lipid packing sensor (or ALPS) domain is an amphipathic helix that can recognize curved vesicle surfaces with radii of the order of 50 nm [27]. A number of recent studies have investigated the curvature-mediated absorption of N-BAR domains onto tubular membranes drawn from giant unilamellar membranes [29–31]. While a detailed analysis of these systems lies beyond the scope of this paper, we note that these surfaces are far more curved than the cellular curvature in bacteria (*r* ∼ 1 *μ*m), indicating again that protein clustering likely plays an important role for subcellular localization in bacteria. Traditionally, entirely different mechanisms for curvature sensing have been invoked for amphipathic helices and BAR domains in the literature. However, Bhatia et al. [32, 33] have argued that amphipathic helices associated with BAR proteins (N-BAR domains) mediate both membrane binding and curvature sensing. Thus a unified model of membrane curvature detection centered on amphipathic helices has been suggested [3, 33]. Thus, given the ubiquity of curvature sensing by amphipathic helices, our analysis, suitably modified, could have widespread significance for protein localization and curvature sensing both in prokaryotic and eukaryotic cells.

## Supporting Information

S1 File Supporting calculational details, Figs. S1–S5. (PDF)

## References

[1] Gitai Z (2009) New fluorescence microscopy methods for microbiology: sharper, faster, and quantitative. Curr Opin Microbiol 12: 341–346. 10.1016/j.mib.2009.03.001 19356974 PMC2741158

[2] Shapiro L, McAdams HH, Losick R (2009) Why and how bacteria localize proteins. Science 326: 1225–1228. 10.1126/science.1175685 19965466 PMC7531253

[3] Huang KC, Ramamurthi KS (2010) Macromolecules that prefer their membranes curvy. Mol Microbiol 76: 822–832. 10.1111/j.1365-2958.2010.07168.x 20444099 PMC2909655

[4] van Ooij C, Losick R (2003) Subcellular localization of a small sporulation protein in Bacillus subtilis. J Bacteriol 185: 1391–1398. 10.1128/JB.185.4.1391-1398.2003 12562810 PMC142862

[5] van Ooij C, Eichenberger P, Losick R (2004) Dynamic patterns of subcellular protein localization during spore coat morphogenesis in Bacillus subtilis. J Bacteriol 186: 4441–4448. 10.1128/JB.186.14.4441-4448.2004 15231775 PMC438564

[6] Ramamurthi KS, Lecuyer S, Stone HA, Losick R (2009) Geometric cue for protein localization in a bacterium. Science 323: 1354–1357. 10.1126/science.1169218 19265022 PMC2652684

[7] Huang KC, Mukhopadhyay R, Wingreen NS (2006) A curvature-mediated mechanism for localization of lipids to bacterial poles. PLoS Comput Biol 2: e151 10.1371/journal.pcbi.0020151 17096591 PMC1635540

[8] Mukhopadhyay R, Huang KC, Wingreen NS (2008) Lipid localization in bacterial cells through curvature mediated microphase separation. Biophys J 95: 1034–1049. 10.1529/biophysj.107.126920 18390605 PMC2479595

[9] Ursell T, Huang KC, Peterson E, Phillips R (2007) Cooperative gating and spatial organization of membrane proteins through elastic interactions. PLoS Comput Biol 3: e81 10.1371/journal.pcbi.0030081 17480116 PMC1864995

[10] Weikl TR, Kozlov MM, Helfrich W (1998) Interaction of conical membrane inclusions: Effect of lateral tension. Phys. Rev. E 57: 6988–6995. 10.1103/PhysRevE.57.6988

[11] Goulian M, Bruinsma R, Pincus P (1993) Long-Range Forces in Heterogeneous Fluid Membranes. Europhys Lett 22: 145 10.1209/0295-5075/22/2/012

[12] Groenewold J, Kegel WKJ (2001) Anomalously Large Equilibrium Clusters of Colloids. J Phys Chem B 105: 11702–11709. 10.1021/jp011646w

[13] Segre N, Prasad V, Schooald AB, Weitz DA (2001) Glasslike Kinetic Arrest at the Colloidal-Gelation Transition. Phys Rev Lett 86: 6042–6045. 10.1103/PhysRevLett.86.6042 11415424

[14] Sciortino F, Mossa S, Zaccarelli E, Tartaglia P (2004) Equilibrium Cluster Phases and Low-Density Arrested Disordered States: The Role of Short-Range Attraction and Long-Range Repulsion. Phys Rev Lett 93: 055701 10.1103/PhysRevLett.93.055701 15323710

[15] Stradner A, Sedgwick H, Cardinaux F, Poon WCK, Egelhaaf SU, et al (2004) Equilibrium cluster formation in concentrated protein solutions and colloids. Nature 432: 492–495. 10.1038/nature03109 15565151

[16] Destainville N (2008) Cluster Phases of membrane proteins. Phys. Rev E 77: 011905 10.1103/PhysRevE.77.011905 18351874

[17] Evans AR, Turner MS, Sens P (2003) Interactions between proteins bound to biomembranes. Phys. Rev. E. 67: 041907 10.1103/PhysRevE.67.041907 12786396

[18] Sieber JJ, Willig K, Kutzner C, Gerding-Reimers C, Harke B, et. al (2007) Anatomy and Dynamics of a Supramolecular Membrane Protein Cluster. Science 317: 1072–1077. 10.1126/science.1141727 17717182

[19] Destainville N, Foret L (2008) Thermodynamics of nanocluster phases: A unifying theory. Phys Rev E 77: 051403 10.1103/PhysRevE.77.051403 18643067

[20] Gurry T, Kahramanogullari O, Endres RG (2009) Biophysical mechanism for Ras-nanocluster formation and signaling in plasma membrane. PLoS ONE 4: e6148 10.1371/journal.pone.0006148 19587789 PMC2704371

[21] Meilhac N., Destainville N. (2011) Clusters of proteins in biomembranes: Insights into the roles of interaction potential shapes and of protein diversity. J Phys Chem B 115: 7190–7199. 10.1021/jp1099865 21528886

[22] Phillips R, Kondev J (2008) Physical Biology of the Cell (Garland Science).

[23] Joachim OR, Feder TJ, Strey HH, Sackmann E (1995) Fluctuation analysis of tension-controlled undulation forces between giant vesicles and solid substrates. Phys Rev E 51: 4526–4536. 9963165 10.1103/physreve.51.4526

[24] Loeffler JM, Nelson D, Fischetti VA (2001) Rapid killing of Streptococcus pneumoniae with a bacteriophage cell wall hydrolase. Science 294: 2170–2172. 10.1126/science.1066869 11739958

[25] Daniel A, Euler C, Collin M, Chahales P, Gorelick KJ, Fischetti VA (2010) Synergism between a novel chimeric lysin and oxacillin protects against infection by methicillin-resistant Staphylococcus aureus. Antimicrob Agents Chemother 54:1603–1612. 10.1128/AAC.01625-09 20086153 PMC2849374

[26] Fischetti VA (2005) Bacteriophage lytic enzymes: novel anti-infectives. Trends Microbiol 13: 491–496. 16125935 10.1016/j.tim.2005.08.007

[27] Bigay J, Casella JF, Drin G, Mesmin B, Antonny B (2005) ArfGAP1 responds to membrane curvature through the folding of a lipid packing sensor motif. Embo J 24: 2244–2253. 10.1038/sj.emboj.7600714 15944734 PMC1173154

[28] Peter BJ, Kent HM, Mills IG, Vallis Y, Butler PJ, Evans PR, McMahon HT (2004) BAR domains as sensors of membrane curvature: the amphiphysin BAR structure. Science 303: 495–499. 10.1126/science.1092586 14645856

[29] Sorre B, Callan-Jones A, Manzi J, Goud B, Prost J, et al (2011) Nature of curvature coupling of amphiphysin with membranes depends on its bound density. Proc Natl Acad Sci USA 109:173–178. 10.1073/pnas.1103594108 22184226 PMC3252917

[30] Singh P, Mahata P, Baumgart T, Das SL (2012) Curvature sorting of proteins on a cylindrical lipid membrane tether connected to a reservoir. Phys Rev E 85: 051906 10.1103/PhysRevE.85.051906 23004787 PMC3679408

[31] Zhu C, Das SL, Baumgart T (2012) Nonlinear Sorting, Curvature Generation, and Crowding of Endophilin N-BAR on Tubular Membranes. Biophys J 102: 1837–1845. 10.1016/j.bpj.2012.03.039 22768939 PMC3328703

[32] Bhatia VK, Madsen KL, Bolinger P, Kunding A, Hedegard P, et al (2009) Amphipathic motifs in BAR domains are essential for membrane curvature sensing. EMBO J 28: 3303–3314. 10.1038/emboj.2009.261 19816406 PMC2776096

[33] Bhatia VK, Hatzakis NS, Stamou D (2010) A unifying mechanism accounts for sensing of membrane curvature by BAR domains, amphipathic helices and membrane-anchored proteins. Semin Cell Dev Biol 21: 381–390. 10.1016/j.semcdb.2009.12.004 20006726

