# Supplementary material for: Modeling Curvature-Dependent Subcellular Localization of the Small Sporulation Protein SpoVM in Bacillus subtilis
Source: PLoS One. 2015 Jan 27;10(1):e0111971. doi: 10.1371/journal.pone.0111971 (PMC4307972; doi:10.1371/journal.pone.0111971)
Supplement: S1 File — (PDF) [file pone.0111971.s001.pdf]

Supporting Information for *Modeling curvature-dependent subcellular localization of the small sporulation protein SpoVM in Bacillus subtilis*

Absorption profiles in the absence of repulsion

In this section we will demonstrate the necessity of including repulsive interactions in order to fit the experimental data for membrane absorption versus SpoVM concentration (see Fig. 4, main text). In the absence of repulsive interactions, with increasing SpoVM concentration we find an abrupt transition to vesicles saturated with SpoVM. In the absence of repulsion the only relevant energetics governing SpoVM absorption are nearest neighbor attraction as well as the binding energy of individual proteins on the membrane. With this in mind we can reduce the problem to an Ising model. The energetics of SpoVM on the vesicle can be given by

$$E = \epsilon \sum_{<i,j>} \sigma_i \sigma_j \tag{S1}$$

where, $< i, j >$ indicates a sum over nearest neighbor lattice sites on the vesicle. σ_i is assumed to be 1 if a lattice site is occupied and 0 otherwise. Let v be the total number of sites on the vesicle and n be the average occupancy per site of the vesicle. In the standard mean field approximation [1], the partition function of the system can be written as

$$Z = e^{-\beta N \epsilon n^2 z/2}(1 + e^{\beta(\epsilon n z + \mu)})^v, \tag{S2}$$

where $\beta = \frac{1}{k_B T}$, T is the temperature, μ is the chemical potential and z is the number of nearest-neighbor sites to each site on the vesicle. Since the average vesicle occupancy is given by $n = \frac{1}{v}\frac{d \ln Z}{d\mu}$ we obtain the following relation

$$n = \frac{e^{\beta(\epsilon n z + \mu)}}{1 + e^{\beta(\epsilon n z + \mu)}}. \tag{S3}$$

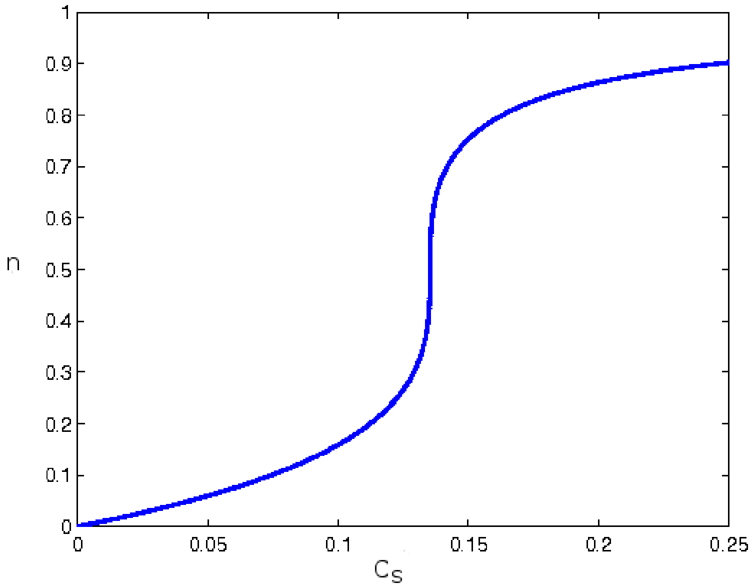

Figure S1: Absorption versus protein molar concentration in solution in the case of zero repulsion.

Chemical potential is related to the concentration of proteins in the solution C_s by the relationship $e^{\mu} = \beta C_s$. Plugging this in the equation above we get

$$n = \frac{e^{\beta \epsilon n z} C_s}{1 + e^{\beta \epsilon n z} C_s} \tag{S4}$$

The plot of n vs C_s for $\beta \epsilon z = 4$ is shown in Fig. S1. While the plot appears to be sensitive to the choice of $\beta \epsilon z$, we note that $\exp(\beta \epsilon n z)$ multiplies C_s in the equation above, and thus can absorbed into C_s. Thus the choice of $\beta \epsilon z$ does not affect the nature of the curve. We find that above a critical concentration the absorption of proteins on the vesicle spikes, implying a phase transition from SpoVM existing as monomers on the smooth surface of the vesicle to SpoVM forming one giant cluster. In order to smoothen this abrupt rise in absorption for increasing SpoVM concentration, repulsive interactions have to be included.

Technical details of the full model derivation

The entropy is

$$\begin{aligned} S &= k_B \ln \frac{L_s!}{(n_s - n_v)!(L_s - n_s + n_v)!} \\ &+ k_B \ln \frac{L_v!}{\Pi_k n_k!(L_v - \sum_k n_k)!} \end{aligned} \tag{S5}$$

where $n_v = \sum_k k n_k$. Using the sterling approximation $\ln N! = N \ln N - N$, the above equation becomes

$$\begin{aligned} S/k_B &= L_s \ln(L_s) \\ &- L_s \\ &- (n_s - n_v)\ln(n_s - n_v) + (n_s - n_v) \\ &- (L_s - n_s + n_v)\ln(L_s - n_s + n_v) \\ &+ (L_s - n_s + n_v) \\ &+ L_v \ln(L_v) - L_v \\ &- \sum_k (n_k \ln(n_k) - n_k) \\ &- (L_v - \sum_k m_k)\ln(L_v - \sum_k n_k) \\ &+ (L_v - \sum_k n_k) \end{aligned} \tag{S6}$$

$$\tag{S7}$$

implying

$$\begin{aligned} S/k_B &= L_s \ln(L_s) \\ &- (n_s - n_v)\ln(n_s - n_v) \\ &- (L_s - n_s + n_v)\ln(L_s - n_s + n_v) \\ &+ L_v \ln(L_v) \\ &- \sum_k m_k \ln(m_k) \\ &- (L_v - \sum_k m_k)\ln(L_v - \sum_k m_k) \end{aligned} \tag{S8}$$

$$\tag{S9}$$

where $n_V = \sum_k k m_k$. The free energy is given by

$$F = E - TS \tag{S10}$$

In order to obtain the equilibrium distribution of cluster sizes, we minimize F and set $\frac{\partial F}{\partial m_k} = 0$. This gives us

$$\begin{aligned}\frac{\partial E}{\partial m_k} &= -k_B T[-k \ln(n_s - \sum k m_k) - k] \\ &- k_B T[k \ln(L_s - n_s + n_v) + k] \\ &- k_B T[\ln(m_k) + 1] \\ &- k_B T[-\ln(L_v - \sum m_k) - 1], \end{aligned} \tag{S11}$$

implying

$$\begin{aligned}\frac{\partial E}{\partial m_k} &= k_B T[k \ln \frac{n_s - n_v}{L_s - n_s + n_v} \\ &+ \ln \frac{L_v - \sum m_k}{m_k}]. \end{aligned} \tag{S12}$$

Defining $n_s^* = \frac{n_s}{L_s}$, this can be written as

$$\begin{aligned}\frac{\partial E}{\partial m_k} &= k_B T[k \ln \frac{n_s^*}{1 - n_s^*} \\ &+ \ln \frac{L_v - \sum m_k}{m_k}]. \end{aligned}$$

If $\sum m_k << L_v$ and $n_s^* << 1$, we have

$$\begin{aligned}\frac{\partial E}{\partial m_k} &= k_B T[k \ln(n_s^*) \\ &+ \ln \frac{L_v}{m_k}], \end{aligned} \tag{S13}$$

and hence,

$$m_k = L_v (n_s^*)^k e^{-\frac{1}{k_B T} \frac{\partial E}{\partial m_k}}. \tag{S14}$$

Thus SpoVM absorption can be expressed as

$$n_v = \sum_k k m_k = \sum_k k L_v (n_s^*)^k e^{-\frac{1}{k_B T} \frac{\partial E}{\partial m_k}}. \tag{S15}$$

Since

$$E = -\epsilon_b n_v + \sum_k [-(k-1)\epsilon_{nn} + \frac{1}{2} k(k-1)\epsilon_r] m_k, \tag{S16}$$

eq. (S15) becomes

$$n_v = L_v e^{-\epsilon_{nn}/k_BT} \sum_k k \; e^{-(\frac{k(k-1)\epsilon_r}{2k_BT})} \left(\frac{c_s}{c_0}\right)^k . \tag{S17}$$

where $c_s = \alpha n_s^*$, $c_0 = e^{-(\epsilon_{nn}+e_b)/k_BT}\alpha$, with α being a constant.

If we define the normalized concentration of proteins in the solution, $C_s = c_s/c_0$ and the normalized concentration of proteins bound to the vesicle $C_v = n_v/(L_v e^{-\epsilon_{nn}/k_BT})$, and $e_r = \epsilon_r/k_BT$ we can write

$$C_v = \sum_k k \; e^{\frac{-k(k-1)e_r}{2}} \; (C_s)^k . \tag{S18}$$

Let us evaluate the value of $k = k^*$ corresponding to the term with the largest contribution to the above sum. For this purpose, we have to maximize

$$k \; e^{\frac{-k(k-1)e_r}{2}} \; (C_s)^k , \tag{S19}$$

by setting its derivative with respect to k to zero. This yields

$$\frac{1}{k^*} - (2k^* - 1)\frac{e_r}{2} + \ln C_s = 0 \tag{S20}$$

For sufficiently large k^*

$$k^* \approx \frac{\ln C_s}{e_r} . \tag{S21}$$

We can approximate the largest term contributing to the absorption C_v for large k^*:

$$C_v \approx k^*[e^{-(k^*-1)e_r/2} C_s]^{k^*} \approx k^* C_s^{\frac{k^*}{2}} , \tag{S22}$$

implying

$$\ln C_v \approx \ln \frac{\ln C_s}{e_r} + \ln C_s \frac{\ln C_s}{2e_r} \approx \frac{(\ln C_s)^2}{2e_r} . \tag{S23}$$

Stoichiometric clustering

We now consider the case when the membrane bound SpoVM forms complexes of a specific size. While we have no strong reason to suspect the presence of a stoichiometric complex, we consider this case for completeness. On the membrane we might expect to have monomers and n-mers where n is around 3 based on the date presented in Fig 1 and 4 (main text).

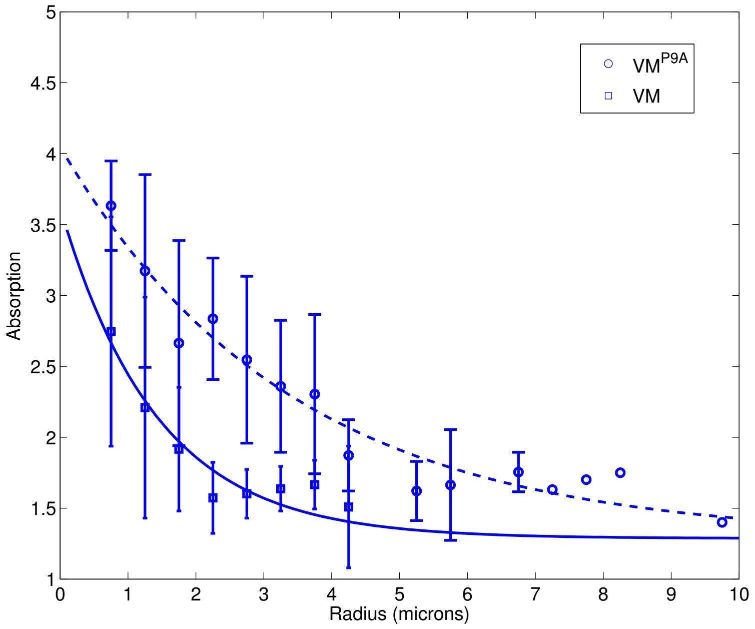

Figure S2: Stoichiometric case: SpoVM absorption vs vesicle radius with $e_r = .20 + .12r$ for VM and $e_r = .18 + .05r$ for VM$^{\text{P9A}}$, assuming only monomers and stoichiometric clusters of size three on the membrane for the surface tension model.

In line with our discussion for the non-stoichiometric case, we assume that the repulsive energy of the n-mers have a radius dependence given by the form $e_r = c_1 + c_2 r$. The absorption of SpoVM on the vesicle in this case can be written as

$$C_v = C_s + 3e^{-\frac{6e_r}{k_B T}} C_s^3 \tag{S24}$$

In Figs. S2 and S3, we plot absorption as a function of vesicle radius and of concentration in solution in this case. While the data is still reasonably well fit by the stoichiometric model, results from the stoichiometric model are expected to deviate sharply from our clustering model if VM concentration is further increased or vesicle radius is further reduced. In Fig. S4 we plot the average cluster size (defined as the total number of membrane-associated VM divided by the total number of clusters) as a function of VM concentration. Again, we see a sharp difference between the two models at higher values of concentration.

Protein absorption and clustering for finite-range repulsion

As discussed in the main text, let us assume that repulsion between membrane-bound SpoVM has a finite range with a sharp cutoff (resembling a step-function). In this case, repulsion energy of a cluster scales as cluster size squared, (k^2), for clusters smaller than a critical size k^*, but increases linearly with cluster size for larger clusters. For a linear cluster, the total energy for $k > k^*$ can be written as

$$\begin{aligned} E &= -\epsilon_{nn}(k-1) + k_{\max}(k_{\max}-1)\epsilon_r + k_{\max}(k-k_{\max})\epsilon_r \\ &= -\epsilon_{nn}(k-1) + k_{\max}(k-1)\epsilon_r \end{aligned} \tag{S25}$$

Hence the total energy associated with membrane bound SpoVM becomes

$$\begin{aligned} E &= -\epsilon_b \sum_k k m_k + \sum_{k=1}^{k=k_{\max}} [-(k-1)\epsilon_{nn} + \frac{1}{2}k(k-1)\epsilon_r] m_k \\ &+ \sum_{k=k_{\max}}^{k=\infty} [-\epsilon_{nn}(k-1) + k_{\max}(k-1)\epsilon_r] m_k, \end{aligned} \tag{S26}$$

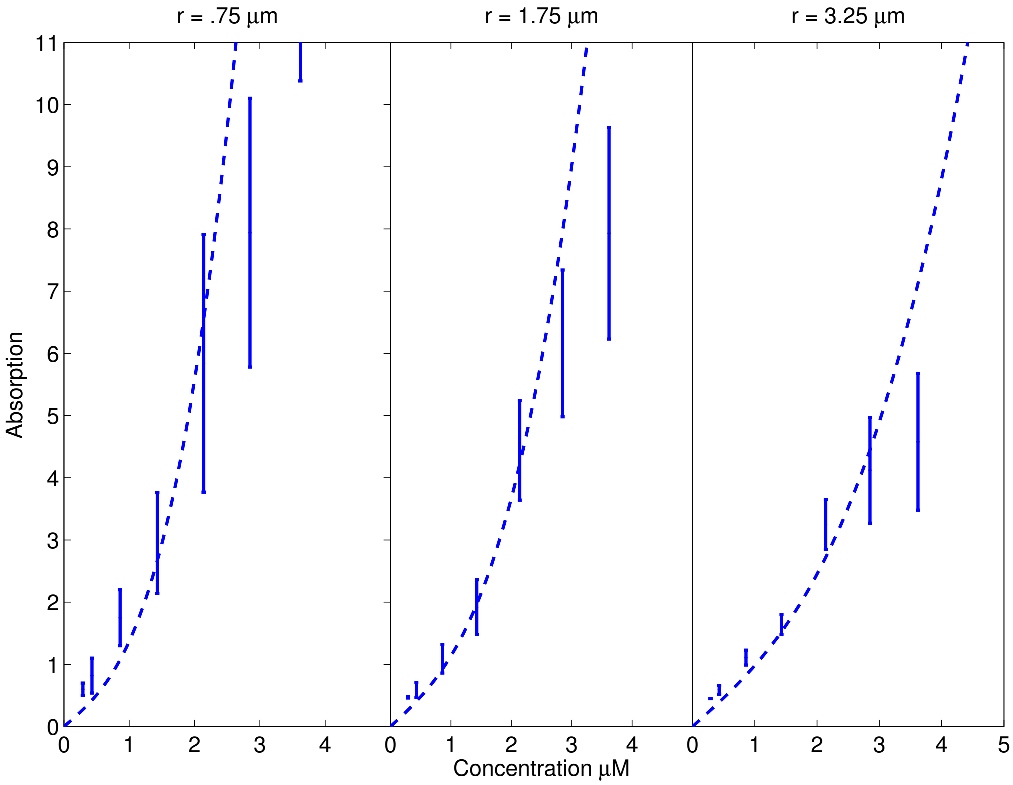

Figure S3: Stoichiometric case: SpoVM absorption versus concentration with $e_r = .20 + .12r$ for VM and $e_r = .18 + .05r$ for VM^{P9A}, for different vesicle radii, assuming only monomers and stoichiometric clusters of size three on the membrane.

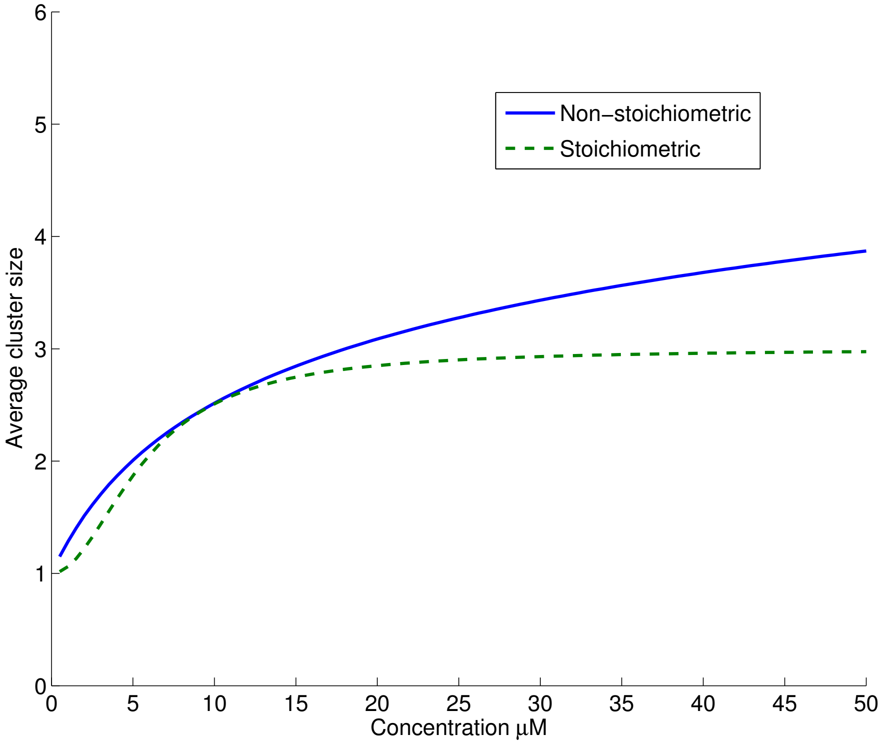

Figure S4: Average cluster size versus concentration for vesicle radius $r = .75\ \mu$m.

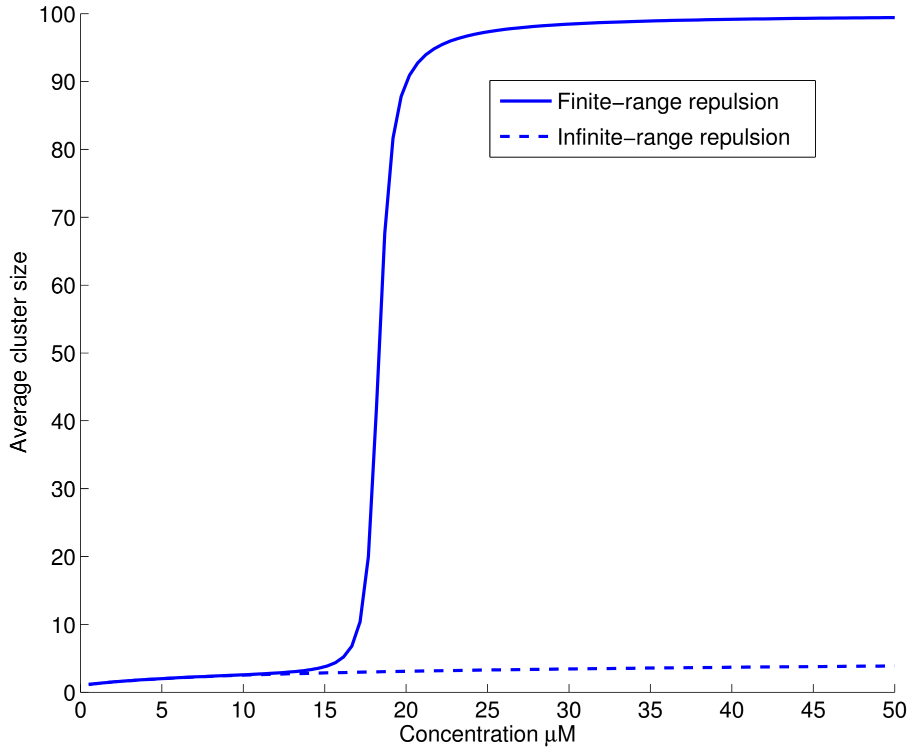

Figure S5: Average cluster size versus concentration of proteins on the vesicle for the finite-range versus infinite-range repulsion for $e_r = .5802$ ($r = .75$ μm from our infinite-range repulsion model that best fits the data). The finite-range repulsion is assumed to be a step function with zero strength for proteins farther apart than fifth nearest neighbor.

Setting $e_r = \epsilon_r/k_BT$, due to the finite-range repulsion Eq (S18) becomes,

$$\begin{aligned} C_v &= \sum_{k=1}^{k=k_{\max}} ke^{-k(k-1)e_r/2}\left(\frac{c_s}{c_0}\right)^k \\ &+ \sum_{k=k_{max+1}}^{k=\infty} ke^{-k_{\max}(k-1)e_r/2}\left(\frac{c_s}{c_0}\right)^k \end{aligned}$$

implying

$$\begin{aligned} C_v &= \sum_{k=1}^{k=k_{\max}} ke^{-k(k-1)e_r/2+ke_rk_{\max}/2}\left(\frac{c_s}{c_0e^{e_rk_{\max}/2}}\right)^k \\ &- e^{e_rk_{\max}}\sum_{k=1}^{k=k_{\max}} k\left(\frac{c_s}{c_0e^{e_rk_{\max}/2}}\right)^k \\ &+ e^{e_rk_{\max}/2}\sum_{k=1}^{k=\infty} k\left(\frac{c_s}{c_0e^{e_rk_{\max}/2}}\right)^k . \end{aligned} \tag{S27}$$

Let us define $x = \left(\frac{c_s}{c_0e^{e_rk_{\max}/2}}\right)$. The above equation becomes

$$\begin{aligned} C_v &= \sum_{k=1}^{k=k_{\max}} ke^{-[k(k-1)e_r+ke_rk_{\max}]/2}x^k \\ &- e^{e_rk_{\max}/2}\sum_{k=1}^{k=k_{\max}} kx^k \\ &+ e^{e_rk_{\max}/2}\sum_{k=1}^{k=\infty} kx^k \end{aligned} \tag{S28}$$

which equals,

$$\begin{aligned} C_v &= \sum_{k=1}^{k=k_{\max}} ke^{-[k(k-1)e_r+ke_rk_{\max}]/2}x^k \\ &- e^{e_rk_{\max}}\sum_{k=1}^{k=k_{\max}} kx^k \\ &+ e^{e_rk_{\max}/2}\frac{x}{(1-x)^2} \end{aligned} \tag{S29}$$

We can easily see that as x approaches 1, C_v diverges implying that SpoVM absorption on the membrane reaches maximum capacity. Thus, as discussed in the text, in the case where we have two membrane regions with slightly different curvatures and, consequently, binding energies, the values of c_0 and hence of x will be slightly different in the two regions. Thus, as x approaches 1 in the region with the higher curvature corresponding to a slightly higher binding energy, the value of x remains smaller than 1 in the other region, leading to pronounced SpoVM localization.

References

[1] Pathria R.K. (1996) *Statistical Mechanics* (Butterworth-Heinemann, 2nd Ed.).
